# Measurement of sulfur L_2,3_ and carbon K edge XANES in a polythiophene film using a high harmonic supercontinuum

**DOI:** 10.1063/1.4964821

**Published:** 2016-10-17

**Authors:** A. S. Johnson, L. Miseikis, D. A. Wood, D. R. Austin, C. Brahms, S. Jarosch, C. S. Strüber, P. Ye, J. P. Marangos

**Affiliations:** Blackett Laboratory, Imperial College London, Prince Consort Road, London SW7 2AZ, United Kingdom

## Abstract

We use a high harmonic generated supercontinuum in the soft X-ray region to measure X-ray absorption near edge structure (XANES) spectra in polythiophene (poly(3-hexylthiophene)) films at multiple absorption edges. A few-cycle carrier-envelope phase-stable laser pulse centered at 1800 nm was used to generate a stable soft X-ray supercontinuum, with amplitude gating limiting the generated pulse duration to a single optical half-cycle. We report a quantitative transmission measurement of the sulfur *L*_2,3_ edge over the range 160–200 eV and the carbon *K* edge from 280 to 330 eV. These spectra show all the features previously reported in the XANES spectra of polythiophene, but for the first time they are measured with a source that has an approximately 1 fs pulse duration. This study opens the door to measurements that can fully time-resolve the photoexcited electronic dynamics in these systems.

## I. INTRODUCTION

X-ray absorption fine structure (XAFS) spectroscopy and X-ray absorption near edge structure (XANES) spectroscopy are well established X-ray spectroscopic techniques that offer insight into the chemical and structural properties of matter. They are widely applied to the study of the static electronic properties of systems ranging from gas phase molecules to solids.^1^ Moreover, the elemental specificity inherent to these techniques offers the possibility of observing the localized electronic structure of the material. Recently, the emergence of time-resolved capabilities from femtosecond beamlines at synchrotrons^2,3^ and free-electron lasers^4,5^ has demonstrated the possibility to study photo-excited dynamics using XANES in pump-probe studies. However, time resolutions much better than 100 fs have been precluded due to the intrinsic pulse duration (in the case of synchrotron sources) and the temporal jitter with respect to laser pump sources (in the case of free electron lasers). In contrast, high harmonic generation (HHG) based sources driven by near-IR to mid-IR lasers have recently been shown to be capable of generating femtosecond timescale pulses into the soft X-ray (SXR) range.^6–8^ As these SXR pulses are precisely synchronized to the laser fundamental field phase and so to other frequency converted pulses derived from the fundamental, there is also the potential offered by this approach for pump-probe measurements with sub-femtosecond temporal resolution.

There are many examples of ultrafast photoexcited dynamics that may be appropriate for study by HHG sources. Here, we discuss developments towards measurements with HHG sources of the ultrafast dynamics in opto-electronic materials such as those used in organic photovoltaics (OPV). In these systems, the efficiency of charge separation is dependent on the dynamics of tightly bound Frenkel excitons^9^ taking place in the first few hundred femtoseconds after they are excited. Excitons have a transitory character and rapidly evolve through a variety of processes including cooling, decay, decoherence through coupling to other particles and lattice phonons, fusion/fission, as well as various localization and transport phenomena.^10^ These processes can lead to effects unfavorable to the functionality of opto-electronic materials, and a fuller understanding of these critical mechanisms through time-domain observations may identify pathways that mitigate these detrimental effects.

Thiophene based organic polymers are widely studied and applied in plastic electronics for the fabrication of photovoltaic devices and field effect transistors. Among the many derivatives of polythiophene, poly(3-hexylthiophene) (P3HT) is the most investigated due to its favorable physical and electronic properties, including high electron mobility in appropriate regioisomers. Its success paved the way for plastic electronics, but further advances with OPV based upon this material are reliant on the development of a deeper understanding of the ultrafast electronic and exciton dynamics in order to identify effective routes to improve the efficiency. Ultrafast studies of these materials have been numerous,^11–15^ and recent research has revealed that critical exciton decay processes occur in less than 50 fs,^16,17^ but this is at the limit of temporal resolution for many ultrafast techniques using optical lasers. Only a handful of studies have approached the 20 fs temporal resolution limit.^16^ As a consequence, little is yet known regarding the initial formation and dynamics of excitons on timescales faster than 10 fs. Moreover, there is a need to measure not just the exciton dynamics but also the localization of the accompanying electronic excitation, which is not accessible using standard techniques. XANES spectroscopy using HHG sources provides a tool potentially capable of measuring exactly these properties, and for these reasons, we are interested in developing time-resolved X-ray spectroscopy methods that can return information on the instantaneous electronic states with, in principle, few-fs temporal resolution. We report here on first steps to achieve these goals, namely demonstrating the generation of a sub-fs duration SXR supercontinuum. We show that with this tool we can probe the static absorption spectra of the sulfur *L*_2,3_ edge and the carbon *K* edge of a P3HT film. Our work is the first to present XANES measurements at two different SXR absorption edges with an HHG-based source. Furthermore, due to source improvements, our spectra are acquired ≈5 times faster than previous XANES spectra acquired with HHG attosecond transients, a key capability for pump-probe studies in which long-term drifts and pump-pulse induced optical damage must be avoided.

This paper is organized as follows. We will first present the experimental methods we use to generate intense few-cycle pulses centered at 1800 nm with stabilized carrier-envelope phase (CEP), and how they are used in generating HHG SXR supercontinua covering the sulfur *L*_2,3_ edge and carbon *K* edge. We restrict the purview of the discussion to those features of the source essential to XANES measurements in P3HT. A technical discussion of the source will be presented in a forthcoming manuscript.^18^ We then present our methodology for preparation of P3HT samples amenable to study via transmission XANES spectroscopy and then present the first XANES measurements at the absorption edges in P3HT obtained with a sub-femtosecond SXR supercontinuum. We discuss the salient features of these spectra, and finally discuss the implications of this step to future time-resolved measurements in P3HT and other materials using HHG supercontinua. This study opens the door to future measurements that can fully time-resolve the photoexcited electronic dynamics in a variety of condensed matter systems.

## II. METHODS

**FIG. 1.**
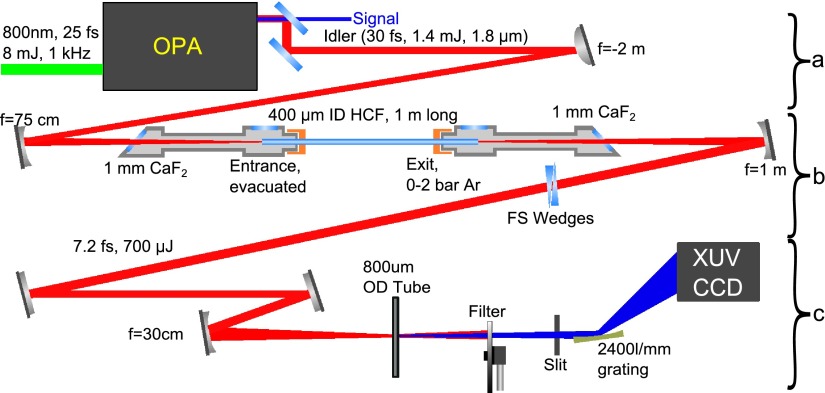
Full system for generating SXR harmonics from a table-top laser source and performing static XANES measurements. The system can be generally described in three parts. (a) Mid-IR pulse generation: the output of our CPA system is passed to a commercial OPA and downconverted. (b) Mid-IR pulse compression: the OPA output is sent through a HCF, spectrally broadened, and compressed in FS wedges. (c) High harmonic SXR supercontinuum generation: the few-cycle mid-IR pulse is focused into a tube filled with Ne and the emitted harmonics are detected in a spectrometer. See text for details.

In most experiments thus far, HHG has been driven by IR pulses centered at 800 nm, owing to the wide availability of modelocked titanium-doped sapphire chirped pulse amplification (CPA) laser systems. While such systems have proved successful^19,20^ in generating isolated attosecond X-ray pulses at energies of up to 110 eV, efforts to extend the photon energies by increasing the on-target intensity have been frustrated by the resulting increase in ionization, which both limits the radiating population and is detrimental to effective macroscopic phase-matching.^21^ In order to circumvent this, we exploit the quadratic scaling with driving wavelength of the HHG cut-off energy^22,23^ and extend our driving wavelength while keeping the intensity below the ionization saturation limit.^24^ As such, we passed the output of our cryogenically cooled, 2-stage Ti:sapph amplifier system, which delivers 800 nm, 8 mJ, 25 fs pulses at 1 kHz, to a commercial white-light seeded 3-stage OPA (HE-TOPAS, Light Conversion Ltd.). We chose to operate the OPA at an idler wavelength of 1.8 *μ*m as a compromise between the cut-off extension and the unfavorable efficiency scaling with wavelength of the single atom response.^25,26^ We obtained 30 fs, 1.4 mJ pulses from our OPA; furthermore, this radiation, being the idler of a self-seeded OPA, is known to be CEP self-stable.^27^ A full diagram of our system, including the HHG generation and detection setup, can be seen in Figure 1.

**FIG. 2.**
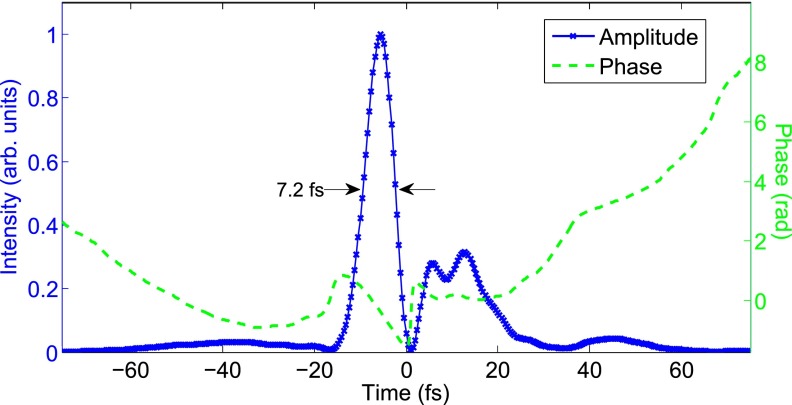
Temporal profile of our few-cycle 1800 nm pulse generated from HCF pulse compression. The intensity (blue crosses) and phase (green-dashed) are both indicated and were determined with SEA-F-SPIDER. The main peak, containing 52% of the energy, has a duration of 7.2 fs FWHM, with the smaller post-pulse having no effect upon the HHG process.

In order to obtain the desired time resolution and to generate isolated harmonic pulses, it was necessary to further temporally compress the idler below two optical cycles. We coupled the 1800 nm pulses into a 1 m long, Ar-filled, differentially pumped hollow-core fiber (HCF). Spectral broadening was achieved through self-phase modulation in the HCF, while the resulting chirp was compensated for by propagation through 3 mm of fused silica (FS), owing to its anomalous dispersion in this wavelength range. The compressed output was measured by a home-built spatially encoded arrangement filter-based spectral phase interferometry for direct electric field reconstruction (SEA-F-SPIDER) setup.^28^ As shown in Figure 2 below, we obtained pulses of around 1.3 optical cycle duration with a total energy of 700 *μ*J. This is sufficiently short for isolated attosecond pulse generation through the amplitude gating mechanism, in which the rapid changes in ionization rate and ponderomotive potential result in isolated harmonic bursts.^29^ Harmonics generated from the post-pulse will not only have a photon energy cut-off three times lower than the main peak, but due to the exponential dependence of the tunneling rate will have an amplitude around 5% that of the main peak. Post-pulse harmonics may be even further suppressed due to ionization gating effects.^30^

**FIG. 3.**
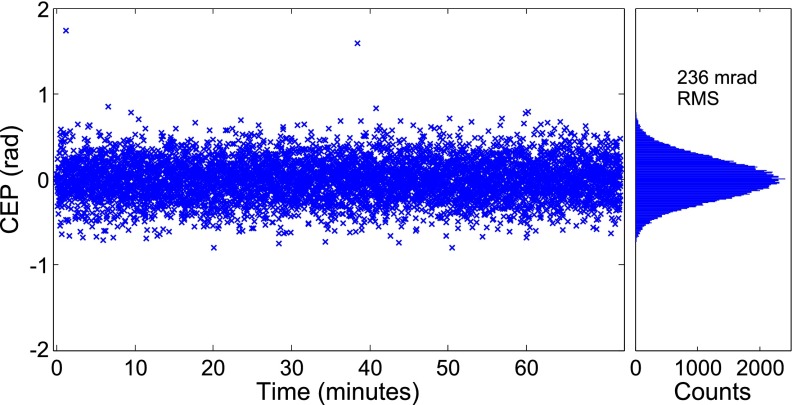
CEP stability of our few-cycle 1800 nm source over 70 min, determined from a 2f-3f interferometry measurement. The CEP stability remains below 250 mrad indefinitely.

Despite the intrinsic CEP stability of a self-seeded OPA, thermal fluctuations result in a slow drift of the average CEP offset. We monitored the CEP using a 2f-3f interferometer and corrected for these drifts with a piezoelectric actuator feedback system which adjusts the pump-seed timing in the OPA. Figure 3 shows the CEP stability of the idler when the feedback loop was active, with a stability of <250 mrad over 1 h typical. This stability is a crucial component of our setup, allowing both the generation of isolated SXR attosecond pulses and the generation of reproducible X-ray spectra for XAFS and XANES measurements.

Employing these 1800 nm, CEP-stable, sub-two cycle driving pulses, we generated SXR high-harmonics in Ne extending well beyond the carbon *K* edge. We focused the 1800 nm beam with an *f* = 30 cm silver coated spherical mirror into a high-pressure gas cell. The target was an 800 *μ*m outer diameter, 720 *μ*m inner diameter tube, which was laser drilled and mounted within a custom two-stage differentially pumped housing, supporting the 1.7 bar backing pressure used in this experiment.^18^ The SXR harmonic spectrum was measured using a spectrometer comprised of a 2400 lines/mm Shimadzu flat-field grating and a water cooled Andor X-ray CCD camera. In order to reject unwanted visible and infrared light, the beam passed through a filter wheel holding a range of thin metallic filters. We also used these filters to calibrate the harmonic spectra using the known absorption edges of various simple compounds. Furthermore, the use of a motorized filter wheel allowed us to easily perform XANES measurements, by switching between the use of a metallic filter and a metallic filter plus the sample. The uniformity of the two metallic filters was seen in the absence of any metallic absorption features in our measured XANES spectra. Using known absorption lines together with ray tracing analysis, we estimate a resolution better than 1 eV at E = 284 eV and 0.5 eV at E = 165 eV.^18^

**FIG. 4.**
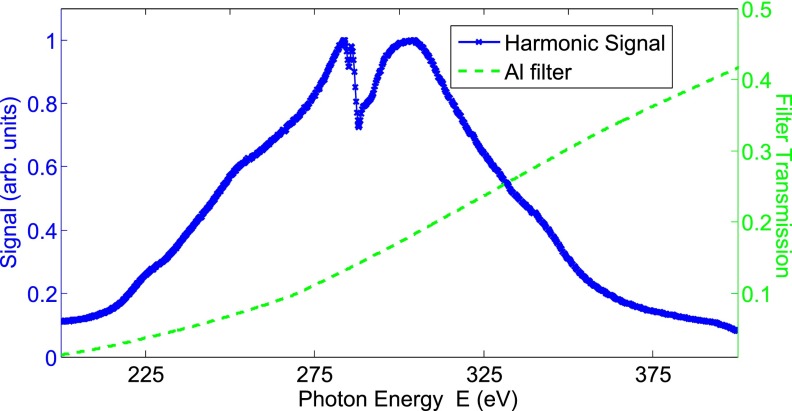
Harmonic spectrum generated in a Ne gas cell by our few-cycle laser source after an Al filter (transmission indicated by the dashed green line). The spectrum does not display any harmonic structure, indicating an isolated harmonic pulse.

Figure 4 shows the HHG spectra generated in Ne used for our XANES measurements on P3HT. Unfortunately, our lack of a suitably broad band-pass filter precludes the measurement of a single spectrum simultaneously spanning all edges of interest. Instead, we use a Zr filter (100–220 eV) at the sulfur *L*_2,3_ edge and an Al filter (200–1600 eV) at the carbon *K* edge. Alternative metallic filters such as In (100–500 eV) would allow simultaneous double-edge-interrogation but were not available at the time of the experiment. The large dip around E = 284 eV results from amorphous carbon contamination on the filters, grating, and XUV camera. Crucially, the existence of a supercontinuum indicates that our XANES measurements are performed with an isolated harmonic pulse. The smooth spectral intensity of the supercontinuum means that the recording of absorption is possible with a constant signal-to-noise ratio over all photon energies in the spectrum, while the extreme bandwidth allows us to probe all features around an edge simultaneously, without the need for scanning. This allows us to, as presented in Section III, obtain a XANES measurement in as little at 2 min.

**FIG. 5.**
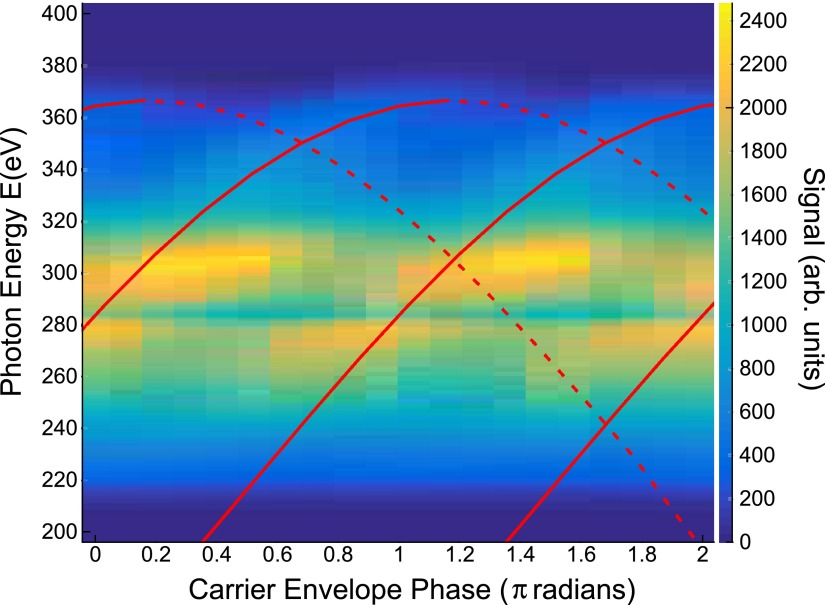
Harmonic spectrum as a function of the CEP of the driving field. Clear half-cycle cut-offs are observable at the highest energies. The red lines are classic trajectory fits to the half-cycle cut-offs which we use to inform an SFA simulation. Dotted red lines indicate trajectories born more than one half cycle before the peak of the field which have weaker amplitude.

The generation of an isolated pulse was further confirmed by observation of CEP dependent harmonic cut-offs within the water window, as shown in Figure 5. For visual clarity, we have corrected for the absorption of the carbon contamination by dividing by the atomic cross section, with residual distortions at the edge attributable to XANES features of the contamination. The CEP of the laser was scanned from 0 to 2*π* and the scan shows the clear *π* periodicity associated with half-cycle cut-off motion. Superimposed over the data are half-cycle cut-offs calculated using classical electron trajectories which best fit the data. Dotted lines indicate trajectories born more than one half cycle before the peak of the field, which have much weaker amplitude than those born around the peak due to reduced tunnel ionization. The high flux at 300 eV is not captured by this simple model and is attributed to phase-matching effects. Further discussion is in the supplementary material. Using a strong-field approximation (SFA) model corresponding to the classical trajectories, we extract an estimate for the temporal structure of our pulse. Further details on the classic trajectories, SFA calculation, and phase-matching effects are in the supplementary material. At energies above 280 eV, we have an isolated pulse of duration 300 as with a bandwidth of 100 eV. At lower photon energies, there are satellite pulses, which do not influence the temporal resolution of measurements at the carbon *K* edge. The 100 eV bandwidth is sufficient to resolve all features of the edge from 280 eV to 330 eV. In the results presented here, the sulfur *L*_2,3_ edge is well below the cutoff, so satellite pulses are possibly significant. Adjusting the cutoff to the lower energy of 165 eV requires only reducing the intensity, with looser focusing an option to mitigate the drop in harmonic flux.

Transmission XANES measurements require the use of thin free-standing films due to the high absorption of X-rays near absorption edges. In a two-colour pump-probe scheme, it is also necessary to balance the target thickness depending on the pump and probe absorption length, in order to maximize the differential signal without introducing unwanted non-linearities or leaving portions of the sample un-pumped. For our planned optical-pump X-ray-probe scheme, we have identified 200 nm thick P3HT films as a good balance of these effects. We manufacture such films in house: Regio-regular poly(3-hexyl)thiophene (rr-P3HT) was first synthesized from 2-bromo-3-hexylthiophene, which was followed by a Soxhlet purification. In order to prepare a deposition substrate, BK7 glass was cleaned with detergent, followed by acetone and isopropanol in a sonication bath, and then treated with an oxygen plasma etch. The substrate was spin coated with 200 nm of poly(4-styrenesulphonic acid) (PSS) to act as a separable layer,^31^ dried, and then spin coated with various concentrations of rr-P3HT in 1,2-dichlorobenzene solution to achieve the desired film thickness. Samples were then thermally annealed in an inert nitrogen atmosphere at 150 °C for 30 min and allowed to cool slowly. The P3HT film was delaminated from the substrate by dissolving the PSS layer and then transferred onto a ring, resulting in 5 mm diameter, 200 nm thick free-standing films. For the thickness measurement, the P3HT film was transferred onto a glass substrate, scratched, and the trench depth measured using a profilometer (Veeco Dektak), with nanometer precision.

**FIG. 6.**
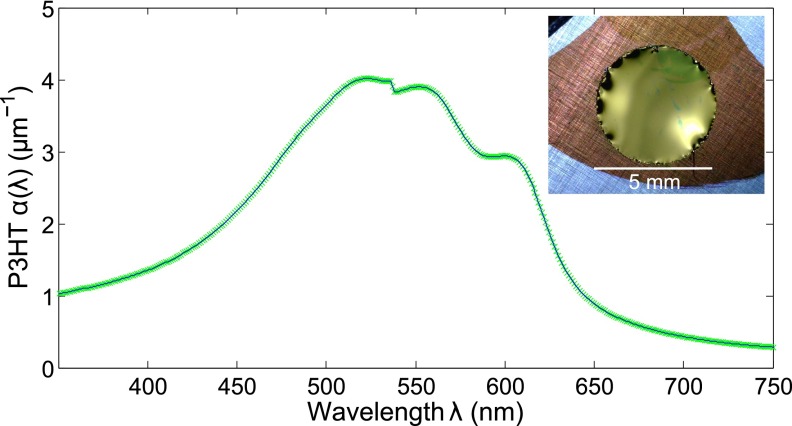
Visible absorption spectrum of our 200 nm free-standing polythiophene films. Inset: Photo of the corresponding film showing the high uniformity.

In order to test the quality of our films, we first performed an optical absorption measurement on our 200 nm, free-standing, annealed P3HT film at normal incidence using a spectrophotometer (Hamamatsu). We use the convention $I_{T}=I_{0}e^{-\alpha d}$, where *I_T_* is the transmitted light, *I*_0_ is the incident light, *α* is the inverse absorption length, and *d* is the sample thickness. Figure 6 shows the obtained absorbance curve. The observed spectrum is in good agreement with typical optical absorption measurements of P3HT films used in OPVs.^32^

## III. RESULTS

XANES measurements were performed by mounting the P3HT film along with a metallic filter (Zr or Al) in the spectrometer prior to the grating. The metallic filter, in addition to blocking visible scatter to the XUV camera, prevents unwanted optical pumping or damage from the fundamental laser pulse. For referencing, a second metallic filter with nominally identical properties as the first, but without a P3HT film co-mounted, was employed. Measurements were performed by acquiring spectra through the filters with and without the sample, integrating for 1 min at each position. All XANES features of interest at the carbon *K* edge could be resolved in the first 2 min of measurement, an ≈5 times shorter acquisition time than previously reported carbon *K* edge measurements with an HHG-based source.^7^ This improvement is due to higher HCF power and looser HHG focusing conditions. Spectra acquired in 2 min can be found in the supplementary material. However, in order to obtain the high signal-to-noise ratio which we present here, we integrated for 1.5 h. This high signal-to-noise ratio with rapid acquisition times will be essential in future time-resolved studies in which dynamical changes may be extremely small. We anticipate further optimization in HHG and in the beam transport/detection will enable significant improvements in data collection times.

**FIG. 7.**
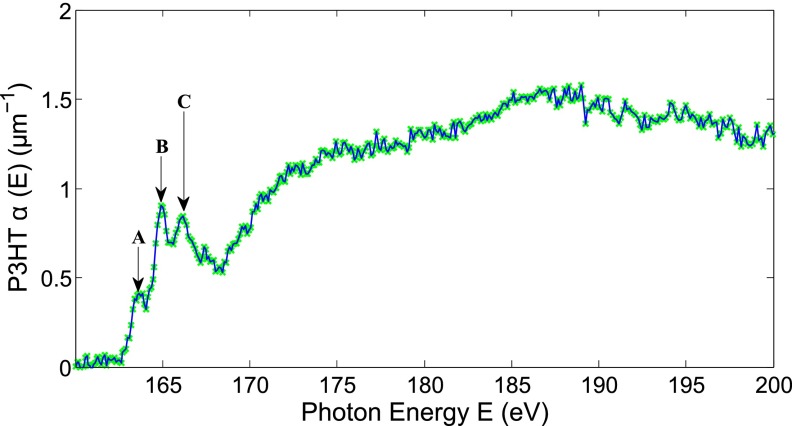
Polythiophene sulfur *L*_2,3_ edge XANES spectrum obtained with our high harmonic supercontinuum. The first three transitions are resolved and are enumerated in Table I.

Figure 7 shows our measurement of the XANES spectrum of P3HT at the sulfur *L*_2,3_ edge (165 eV) through a Zr filter. Note that as with standard XANES analysis, the pre-edge exponential structure has been subtracted, but we have not normalized the results, allowing us to extract quantitative absorption coefficients. We have not subtracted the atomic absorption background or the post-edge structure, as would be common in extended X-ray absorption fine structure (EXAFS) analysis. We can clearly identify the first three absorption features, as listed in Table I, which are in agreement with previous studies.^33^ Weaker shape resonances are not resolved as they require atomic background subtraction to be visible. To our knowledge, this represents the first absolutely calibrated cross sections at the sulfur *L*_2,3_ edge in P3HT, as well as the first transmission XANES measurement at this edge. We did not optimize our source for this photon energy range, but increased flux and thus the signal-to-noise ratio in this photon energy range could be obtained by increasing our focal length for the harmonic generation, increasing the focal spot size and number of emitters, with a corresponding decrease in signal at higher energies. We also note that we see no sign of the Zr *M*-edge lines in the XANES spectrum, indicating that the metallic filter used with the sample and the metallic filter used for referencing have good uniformity. We also observed the relatively weak sulfur *L*_1_ edge at E = 230 eV, but because this was between the window of transmission of the Zr and Al filters, we were not able to resolve the fine structures.

**TABLE I.** Assignment of peaks from the polythiophene sulfur *L*_2,3_ edge XANES spectrum. Peaks' assignment taken from Tourillon *et al.*^33^

Peak	Assignment
A	$2p_{3/2}\to 3b_{1}\pi^{*}$
B	$2p_{1/2}\to 3b_{1}\pi^{*}$
	$2p_{3/2}\to \sigma^{*}(C-S)$
C	$2p_{1/2}\to \sigma^{*}(C-S)$

**FIG. 8.**
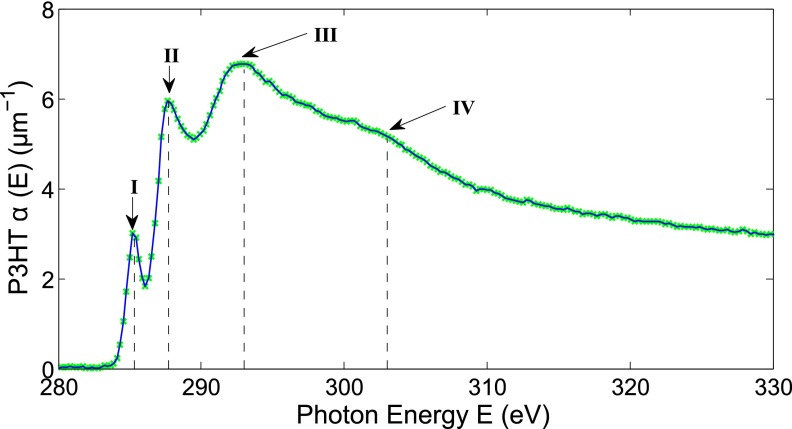
Polythiophene carbon *K* edge XANES spectrum obtained with our high harmonic supercontinuum. All transitions are resolved and are enumerated in Table II.

Figure 8 shows the carbon *K* edge XANES spectrum of our P3HT films; all the relevant transitions are clearly resolved and labelled accordingly as tabulated in Table II, and the agreement with previous measurements performed at synchrotrons is excellent, both qualitatively^33–35^ and quantitatively,^36,37^ assuming a mass density of 1.16 g/cm^3^ in our sample.^38^ The improved signal-to-noise ratio here is due primarily to improved flux, as the generation was optimized for higher photon energies in the data presented, though the superior rejection of lower-order harmonics by the Al filter also contributes.

**TABLE II.** Assignment of peaks from the polythiophene carbon *K* edge XANES spectrum. Peaks' assignment taken from DeLongchamp *et al*.^34^

Peak	Assignment
I	1*s* → 3*b*_1_π^*^
II	$1s\to \sigma^{*}(C-S,\;C-H)$
	$1s\to 2a_{2}\pi^{*}$
III	$1s\to \sigma^{*}(C-C)$
IV	$1s\to \sigma^{*}(C=C)$

## IV. DISCUSSION

The above XANES measurements exhibit several properties which illustrate the potential power of time-resolved XANES studies using harmonic radiation. The measurements exhibit high resolution, clearly resolving all the sharp features known to occur at the sulfur *L*_2,3_ and carbon *K* edges. This is of significant importance, since unlike large scale structural dynamics, excitonic and electronic dynamics are anticipated to introduce small changes to the overall XANES signal. These may manifest as changes in the relative weights of peaks, or even in the emergence of new features as new channels are opened by population transfers. This ties in closely with another useful feature. Because the bandwidth of the harmonic radiation is so large, we simultaneously probe not only the XANES features but also the pre- and post-edge structure over an extended energy range. This allows us to subtract the pre-edge structure from each measurement precisely at no additional cost in acquisition time. Thus, if a new channel were to open below the π^*^ transition, then it would be against a very simple subtractable background. The potential to simultaneously probe multiple edges is of course significant as well; in principle, this technique is scalable to obtain entire XANES measurements in a single sub-femtosecond laser shot. This would allow us also to study stochastic effects, as well as deterministic dynamics. Probing multiple edges allows different channels to be disentangled, as overlapping transitions at one edge may be separable at another.

The use of HHG supercontinua in the SXR region for XANES also brings considerable advantages stemming from the laser-driven origin of the radiation. The HHG supercontinuum is intrinsically synchronized to the driving laser field. If a CEP-stable pulse is used, this synchronization is at attosecond precision. This synchronization has allowed attosecond pulses to be used in a large variety of optical pump-XUV probe experiments,^39,40^ and this intrinsic capability will no doubt be of considerable utility in the SXR region as well. We have, for instance, already used the few-cycle idler pulse for third harmonic generation, which could provide a suitable ≈600 nm source for ultrafast excitation in a pump-probe experiment. Another advantage of HHG is the high coherence of the process when correctly phase-matched. In the time domain, this manifests as the production of attosecond pulses; in the spatial domain, high coherence is also observed, and harmonics are generally emitted as a low divergence beam of laser-like light. This highly coherent, low divergence beam allows for nearly diffraction limited focusing, and the SXR supercontinuum can be efficiently manipulated and coupled to both samples and the measurement apparatus. Further advances in HHG driven by long wavelength fields will additionally enable probing all edges of interest in the water window, particularly at the nitrogen *K* edge and oxygen *K* edge where previous ultrafast studies have revealed exciting dynamical features.^41,42^

## V. CONCLUSIONS

We have demonstrated the generation of an SXR supercontinuum from HHG in Ne driven by a few-cycle mid-IR laser source. The use of a long wavelength source allows the generation of SXR supercontinua spanning hundreds of electron volts, stretching past the carbon *K* edge (284 eV), while the few-cycle CEP-stable nature ensures that the radiation is emitted as a spectrally stable and temporally isolated pulse. HHG supercontinua demonstrate several attractive features for time-resolved XANES measurements, including synchronization to the driving laser, high spatial coherence, and low divergence. We have further demonstrated the utility of this source by performing quantitative XANES measurements at the sulfur *L*_2,3_ and carbon *K* edges of a free-standing P3HT film, yielding the first absolutely calibrated XANES spectra at the sulfur *L*_2,3_ edge. Our work is the first to probe multiple SXR absorption edges with a single harmonic source, and additionally represents the fastest acquisition of a XANES spectrum at the carbon *K* edge with an HHG-source to date. This measurement represents a proof-of-principle experiment for the use of HHG generated SXR supercontinua to perform XANES measurements on OPVs and enables new few-femtosecond resolution time-resolved studies on these systems. Future work will consist of extending these static absorption spectra to studies of ultrafast dynamics via time-resolved XANES spectroscopy of OPVs and other condensed phase targets.

## VI. SUPPLEMENTARY MATERIAL

See supplementary material Section I for details on the classical fitting of the CEP scan, SFA simulation results, and a discussion of phase-matching effects (CEP Scan and SFA Calculation). Carbon *K* edge XANES spectra of P3HT obtained with 2 min of measurement are shown in supplementary material Section II (Two Minute Acquisition of Carbon *K* Edge Spectrum).
